# Supplementary figures and images for: Dogcatcher2: Improved statistical detection of transcriptional readthrough and repetitive element analysis across sequencing platforms
Source: bioRxiv. 2026 May 12:2026.05.07.723642. Preprint. [Version 1] doi: 10.64898/2026.05.07.723642 (PMC13192622; doi:10.64898/2026.05.07.723642)

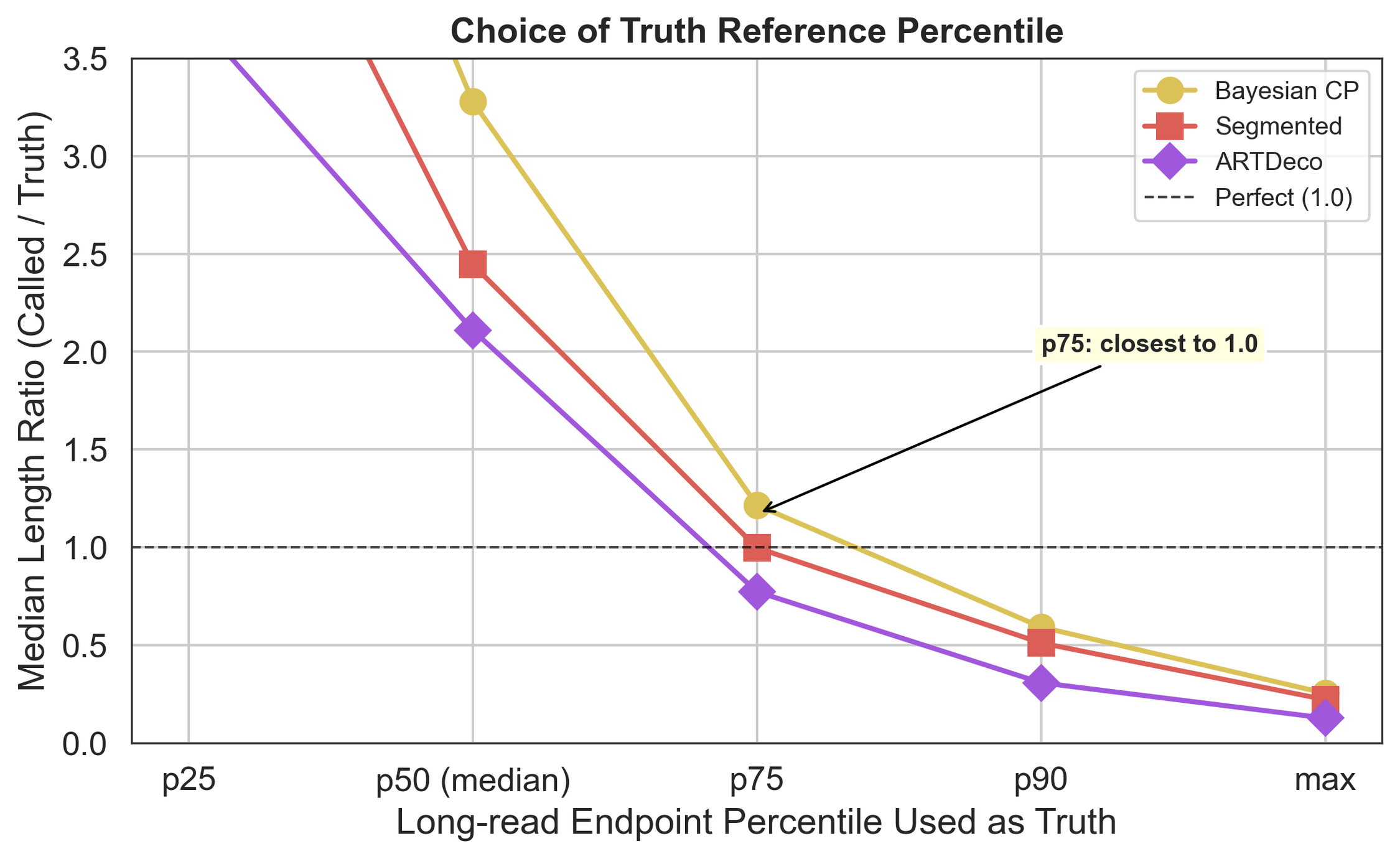

Supplement: Supplement 1 — S1 Fig. Choice of truth reference percentile for boundary accuracy benchmarking. Median length ratio (called DoG length / truth extension) plotted across five long-read endpoint percentiles (p25, p50, p75, p90, max). At the 75th percentile, all three methods (Bayesian CP, Segmented, ARTDeco) converge closest to 1.0, indicating that detector-called boundaries best match the 75th percentile of long-read endpoints. At lower percentiles (p50), detectors overestimate length (ratios 2-3x), while at maximum extension, detectors underestimate (ratios 0.1-0.5). This justifies using the 75th percentile as the truth reference for Boundary F1 calculations throughout the paper. [file media-1.tif]

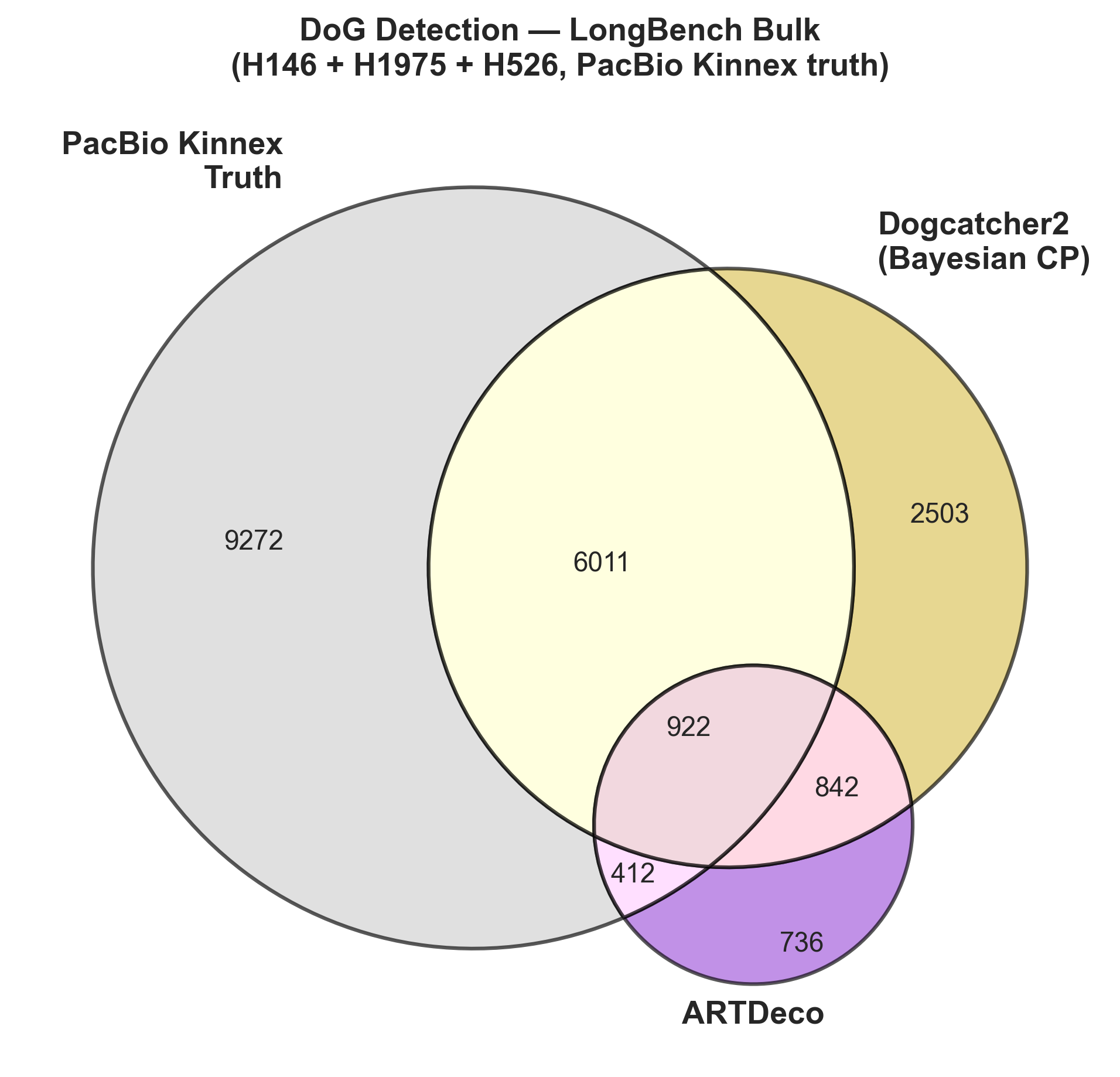

Supplement: Supplement 3 — S2 Fig. Three-way Venn diagram of DoG detection pooled across all three LongBench bulk cell lines (H146 + H1975 + H526). PacBio Kinnex truth identifies 16,617 genes with validated DoGs. Dogcatcher2 (Segmented) detects 10,874 DoGs, of which 7,143 overlap with truth (66% precision). ARTDeco detects 2,912 DoGs with 1,334 overlapping truth (46% precision). 8,932 truth DoGs are missed by both short-read methods, consistent with the depth sensitivity analysis in Fig 4. [file media-3.tif]

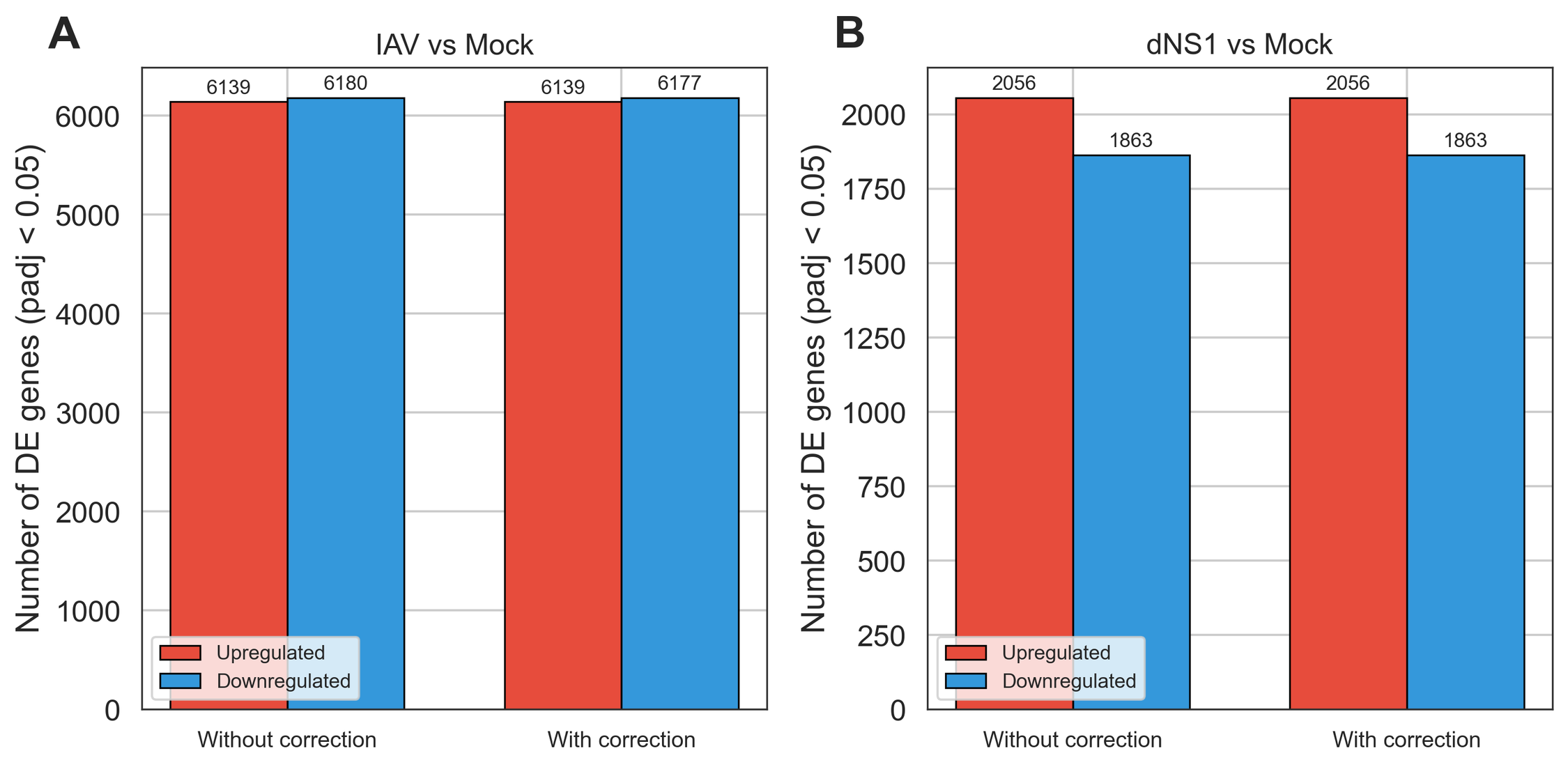

Supplement: Supplement 5 — S3 Fig. Read-in gene correction validation on IAV-infected MDM RNA-seq. Dataset GSE103477 (Heinz et al. 2018), the same dataset used by ARTDeco for validation. IAV infection causes widespread readthrough via NS1 protein inhibiting polyadenylation (CPSF30 inactivation), while dNS1 (NS1-deleted mutant) serves as a control with reduced readthrough. (A) IAV vs Mock: Dogcatcher2’s read-in correction removed 3 spurious DE genes that were falsely called as differentially expressed due to upstream readthrough contaminating their counts. (B) dNS1 vs Mock: minimal readthrough as expected, correction has negligible effect. The small number of corrections is consistent with ARTDeco’s findings, as read-in effects concentrate at a few highly expressed genes near global DoGs, but even a few false positives can mislead pathway enrichment and eQTL interpretation. [file media-5.tif]

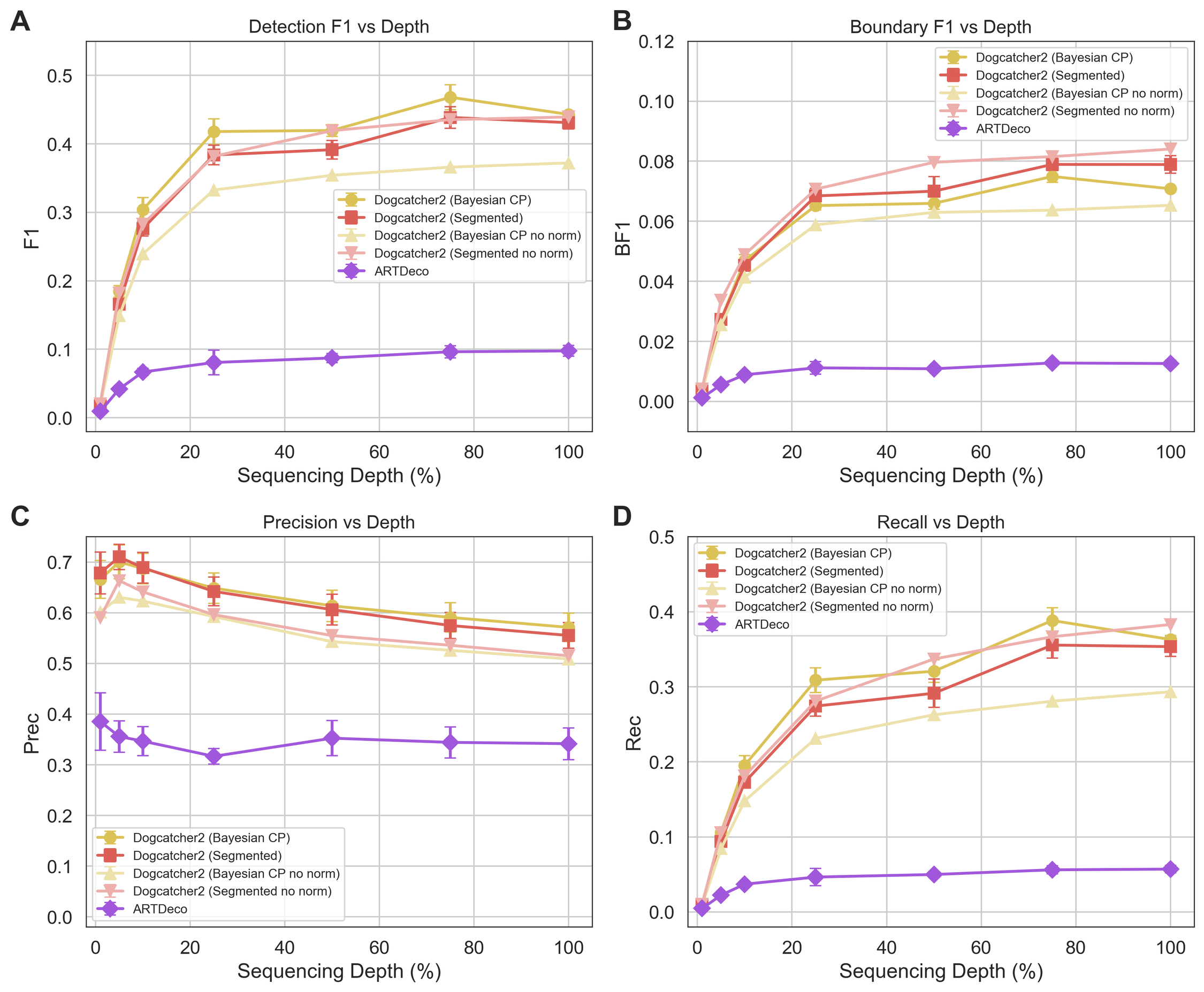

Supplement: Supplement 6 — S4 Fig. Normalization ablation analysis across sequencing depths. Detection F1 (A), Boundary F1 (B), Precision (C), and Recall (D) shown for Dogcatcher2 detectors with and without gene body normalization, compared to ARTDeco, across seven downsampling levels (1-100%). Both Bayesian CP and Segmented detectors outperform ARTDeco even without normalization, demonstrating that the statistical detection methods themselves are the primary source of improvement. Normalization provides an additional 5-15% F1 boost, most pronounced at lower sequencing depths. [file media-6.tif]

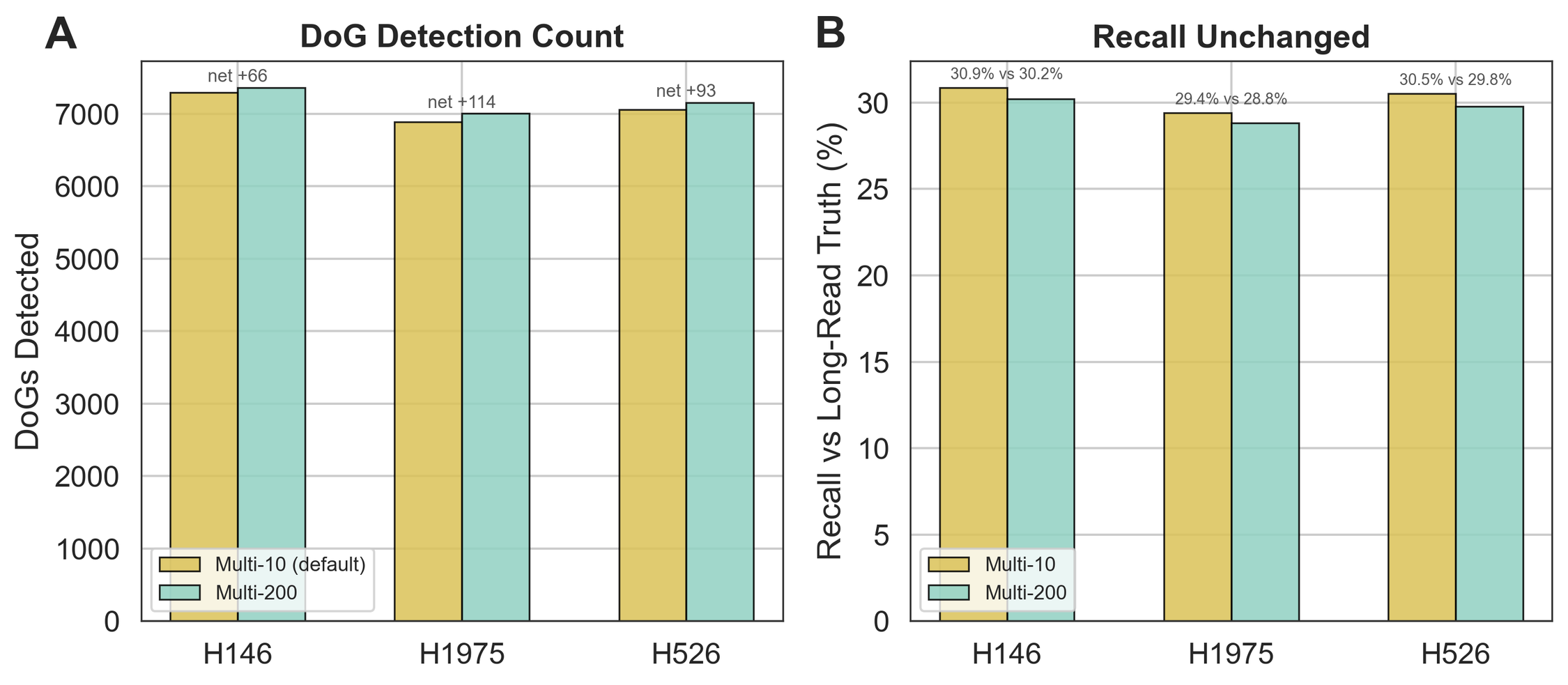

Supplement: Supplement 7 — S5 Fig. Effect of multimapping on DoG detection. Dogcatcher2 (Bayesian changepoint) run on STAR alignments with outFilterMultimapNmax=10 (default) vs outFilterMultimapNmax=200 for all three LongBench cell lines. (A) Multi-200 produced a net gain of only 66-114 DoGs per cell line. (B) Recall vs PacBio Kinnex truth was unchanged or slightly decreased (H146: 30.9% vs 30.2%), demonstrating that the 65% miss rate reflects expression depth limitations, not multimapping artifacts in TE-dense regions. [file media-7.tif]

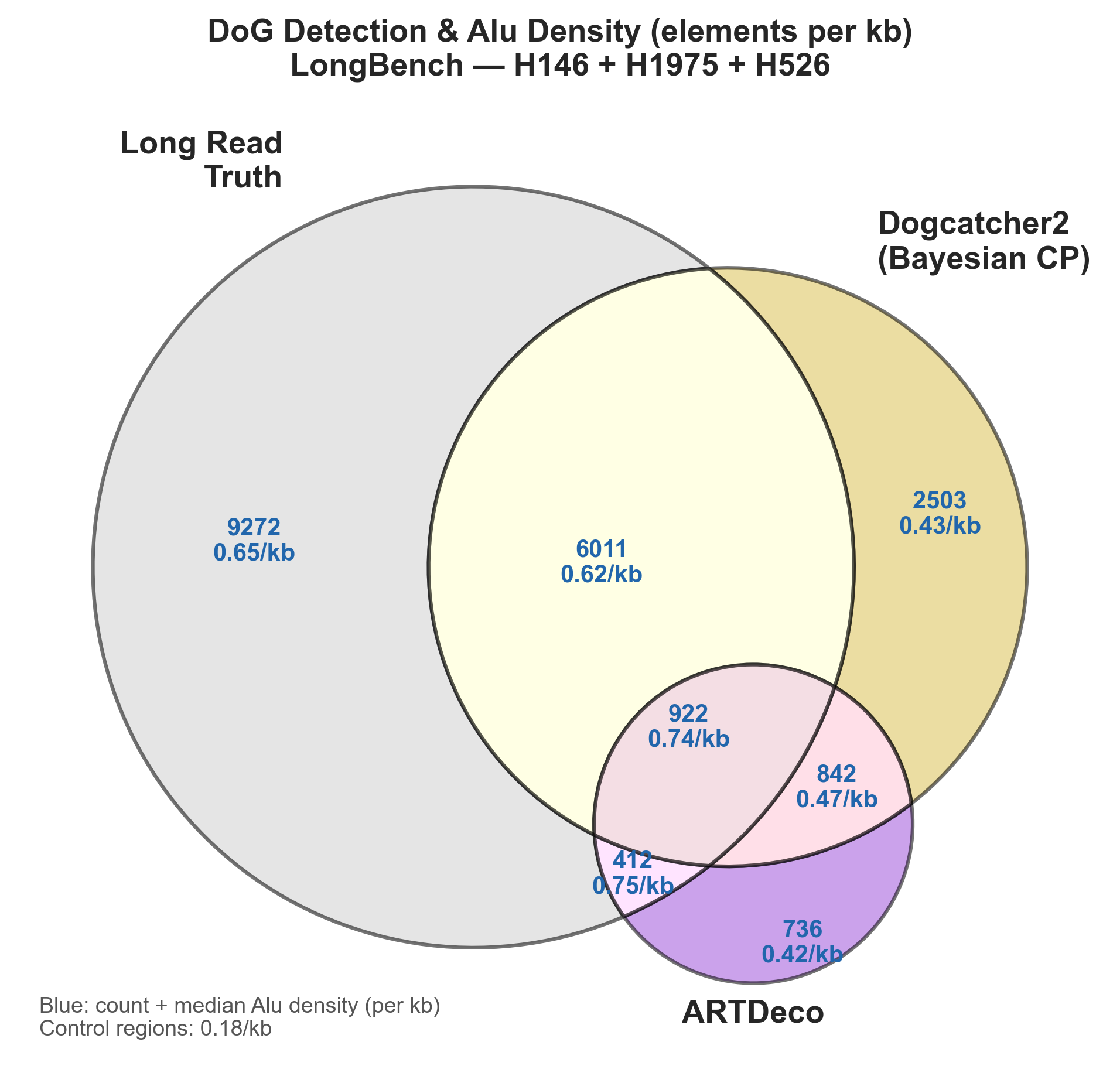

Supplement: Supplement 8 — S6 Fig. Venn diagram of DoG detection with Alu density annotations. Three-way comparison of Long Read Truth, Dogcatcher2 (Bayesian CP), and ARTDeco, with median Alu element density (elements per kb) shown for each section. All DoG regions show elevated Alu density compared to matched non-DoG controls (0.18/kb). Long-read-confirmed regions show 0.62-0.75/kb, while short-read-only DoGs show 0.42-0.47/kb, likely reflecting shorter DoG lengths that sample less Alu-rich downstream sequence. [file media-8.tif]
